# Impact of COVID-19 on the Cardiovascular System: A Review

**DOI:** 10.3390/jcm9051407

**Published:** 2020-05-09

**Authors:** Kensuke Matsushita, Benjamin Marchandot, Laurence Jesel, Patrick Ohlmann, Olivier Morel

**Affiliations:** 1 Pôle d’Activité Médico-Chirurgicale Cardio-Vasculaire, Université de Strasbourg, Nouvel Hôpital Civil, Centre Hospitalier Universitaire, 67091 Strasbourg, France; kmatsushita@unistra.fr (K.M.); benjaminmarchandot@gmail.com (B.M.); Laurence.JESEL-MOREL@chru-strasbourg.fr (L.J.); Patrick.Ohlmann@chru-strasbourg.fr (P.O.); 2 UMR1260 INSERM, Nanomédecine Régénérative, Faculté de Pharmacie, Université de Strasbourg, F-67401 Illkirch, France

**Keywords:** COVID-19, cardiovascular disease, myocardial injury

## Abstract

The recent outbreak of coronavirus disease 2019 (COVID-19) caused by severe acute respiratory syndrome coronavirus 2 has been declared a public health emergency of international concern. COVID-19 may present as acute respiratory distress syndrome in severe cases, and patients with pre-existing cardiovascular comorbidities are reported to be the most vulnerable. Notably, acute myocardial injury, determined by elevated high-sensitivity troponin levels, is commonly observed in severe cases, and is strongly associated with mortality. Therefore, understanding the effects of COVID-19 on the cardiovascular system is essential for providing comprehensive medical care for critically ill patients. In this review, we summarize the rapidly evolving data and highlight the cardiovascular considerations related to COVID-19.

## 1. Introduction

In December 2019, coronavirus disease 2019 (COVID-19) infected pneumonia caused by severe acute respiratory syndrome coronavirus 2 (SARS-CoV-2) occurred in Wuhan, China with further worldwide transmission to a pandemic outbreak. An initial cluster of infections was linked to the Huanan seafood market, potentially due to animal contact [1]. Subsequently, human-to-human transmission occurred, and the disease rapidly spread to people around the globe. As of May 8th 2020, 3,907,055 patients worldwide have been tested positive for COVID-19 with a reported death toll amounting to 272,578 patients, and the numbers continue to rise [2].

SARS-CoV-2 can cause multiple system infections in various animals and mainly respiratory tract infections in humans, such as severe acute respiratory syndrome (SARS) and Middle East respiratory syndrome (MERS) [3]. The majority of patients have mild symptoms, but some patients progress rapidly with acute respiratory distress syndrome (ARDS) and shock, which are followed by multiple organ failure (MOF). Although the typical symptoms in the early stage are fever, fatigue, cough, and muscle ache [3,4], COVID-19 often enters the differential diagnosis of dyspnea at rest and chest pain, which requires engagement of cardiovascular (CV) specialists [5,6]. Moreover, COVID-19 patients with pre-existing cardiovascular disease (CVD) have been reported to be at high risk of adverse outcomes; and infection, itself, is associated with CV complications [7]. Remarkably, recent retrospective studies have demonstrated that acute myocardial injury is a common condition among patients with COVID-19 and is associated with disease severity and mortality [8,9]. Finally, several therapeutics for COVID-19 have the potential for adverse CV side-effects and interactions with CV medications. This review was therefore performed to characterize the epidemiology, clinical features, the direct and indirect impact of COVID-19 on CV systems, and the potential interaction of current available therapies with CV medications and their CV toxicities.

## 2. Pathophysiology of COVID-19

Coronaviruses are enveloped RNA viruses that are distributed broadly among humans and cause respiratory, hepatic, and neurologic disease [10]. Until recently, six different coronavirus strains were known to infect humans. Four viruses, 229E, OC43, NL63, and HKU1, are prevalent and typically cause common cold symptoms in immunocompetent individuals [11]. The two other strains include severe acute respiratory syndrome coronavirus (SARS-CoV) and Middle East respiratory syndrome coronavirus (MERS-CoV), which are zoonotic in origin and have been linked to sometimes fatal illness [11]. SARS-CoV-2 was initially identified from the Chinese city of Wuhan in December 2019 [10]. Like other members of the Coronaviridae family, SARS-CoV-2 is an enveloped virus with a nonsegmented, single stranded, positive-sense RNA genome [8,12]. The SARS-CoV-2 is less genetically similar to MERS-CoV (around 50% nucleotide identity), but has 88% nucleotide identity with bat SARS-like coronavirus and 79% with that human SARS-CoV [13]. The virus is thought to spread mainly from human-to-human through respiratory droplets produced by an infected individual [14]. Studies have demonstrated that SARS-CoV-2, as well as SARS-CoV, binds its viral spike (S) proteins to angiotensin-converting enzyme 2 (ACE2) proteins for cell entry and uses the cellular serine protease transmembrane protease serine 2 (TMPRSS2) for S protein priming [12,15,16,17]. ACE2 is highly expressed in lung alveolar cells (principally type II alveolar cells) and serves a role in lung protection; therefore, viral binding to this receptor deregulates a lung protective pathway, contributing to viral pathogenicity [18,19].

## 3. Epidemiology and Clinical Features of COVID-19

Since December 2019, the disease has spread over 180 countries across the world [2]. As of May 8th 2020, more than 3,900,000 cases have been reported positive for COVID-19, resulting in more than 272,000 deaths. Although the overall fatality rate is 7.0%, it varies wildly depending on age, gender, underlying disease, and country [20]. The fatality rate is considered to be higher than seasonal influenza (less than 0.1%) and lower than SARS (9%) and MERS (36%) [11]. The basic reproduction number (R_0_) of SARS-CoV-2 is estimated to be 2.2, which is greater than that of seasonal influenza (1.3) [21] and MERS-CoV (<1) and similar to that of SARS-CoV (2 to 3) [22]. This estimates that each COVID-19 patient has been spreading infection to 2.2 other people.

Another important reason for the rapid spread is related to the large number of patients with no or mild symptoms with SARS-CoV-2 [4,23]. The asymptomatic proportion was estimated to be 17.9% according to data from people who underwent a two-week quarantine on the Diamond Princess cruise ship [24]. Unlike SARS-CoV, the detected viral load is similar in the asymptomatic and symptomatic patients with COVID-19, which suggests potential transmission of the virus from asymptomatic or mild symptomatic patients to other people [25,26]. Fever is the most common symptom (88.7%) but it can be present in only 43.8% on admission [27]. Since absence of fever in COVID-19 is more frequent than in SARS-CoV (1%) and in MERS-CoV (2%) infection, afebrile patients may be missed if the surveillance case definition only focuses on fever detection [22]. The second most common symptom is cough (67.8%), while nausea or vomiting (5.0%) and diarrhea (3.8%) are uncommon. The median incubation period has been estimated to be four to five days, similar to SARS-CoV [27,28]. According to the retrospective study from China including 191 patients with COVID-19, the median time from illness onset to dyspnea, sepsis, and ARDS were seven days, nine days, and twelve days, respectively [7]. In nonsurvivors, the median time from illness to death was 21 days. Very recently, a cross-sectional survey including 59 patients was reported from Italy, noting that 34% of the patients had olfactory or taste disorders [29]. This may play a role as a clinical screening tool to orientate testing of paucisymptomatic individuals, but larger studies are warranted to confirm this finding.

The most common radiographic findings are ground-glass opacity (56%) and bilateral patch shadowing (52%) on computed tomography (CT) [27]. Electrocardiogram may show nonspecific ST-T abnormalities in some cases [30,31], but the prevalence remains unknown. In laboratory tests, lymphocytopenia is commonly observed (83%) and most patients have elevated serum levels of C-reactive protein and proinflammatory cytokines [7,10]. In particular, patients requiring intensive care unit (ICU) admission had higher concentrations of GCSF, IP10, MCP1, MIP1A, and TNF-α than those not requiring ICU admission, suggesting cytokine storms in critically ill patients [8].

Large case series from China including 72,314 patients with COVID-19 have indicated that the clinical severity was mild (no or mild pneumonia) in 81%, severe (dyspnea, respiratory frequency ≥30/min, blood oxygen saturation ≤93%, partial pressure of arterial oxygen to fraction of inspired oxygen ratio <300, and/or lung infiltrates >50% within 24 to 48 h) in 14%, critical (respiratory failure, septic shock, and/or multiple organ dysfunction or failure) in 5% of the population [32]. In particular, the fatality rate was likely to be higher in patients aged 80 years and older and with pre-existing CVD, diabetes (DM), chronic respiratory disease, hypertension (HT), and cancer [32,33]. Cases aged 70 to 79 years had an 8.0% fatality rate, and cases aged 80 years and older had a 14.8% fatality rate, whereas deaths in children appeared to be very rare [32]. Recent investigation has demonstrated a higher mortality rate in Italy (7.2%) than in China (2.3%), which could be due to the older age distribution in Italy relative to that in China [33]. Importantly, 3.8% of the whole cohort were health workers and 14.8% of those were classified as severe or critical [32]. Lately, increasing numbers of reports from the United States (U.S.) have linked obesity to more severe COVID-19 illness and death [34,35,36].

Currently, no proven specific therapies are available for COVID-19, other than supportive care [37,38]. Available evidence suggests that carefully selected patients with severe ARDS who do not benefit from conventional treatment might be successfully supported with venovenous extracorporeal membrane oxygenation (ECMO) [37,39]. Nevertheless, careful patient selection for ECMO is needed because patient age and comorbidities appear to influence outcomes in critically ill patients with COVID-19. A large number of patients have received off-label and compassionate use therapies such as chloroquine, hydroxychloroquine, lopinavir-ritonavir, remdesivir, ribavirin, favipiravir, interferon, convalescent plasma, steroids, and anti-IL-6 inhibitors, based on either in vitro antiviral or anti-inflammatory properties [38]. However, caution should be taken because no clinical evidence currently supports the efficacy and safety of any drug against any coronavirus in humans, including SARS-CoV-2. Randomized clinical trials (RCTs) have been launched around the world, which may find optimal treatments for COVID-19.

## 4. Cardiovascular Disease in Patients with COVID-19

### 4.1. Prevalence of Cardiovascular Disease in COVID-19 Patients

Previous studies have shown that CVD was a common comorbidity in patients with SARS and MERS. A retrospective case series including 144 patients with SARS examined the prevalence of cardiac disease, DM, and cancer to be 8%, 11%, and 6%, respectively [40]. In MERS, a meta-analysis of 12 studies inclusive of 637 patients indicated that DM and HT were found in 50% of the cases, while CVD was present in approximately 30% of the patients [41]. The higher prevalence of CVD in MERS may be partly explained by the older age distribution in MERS than that in SARS (50.0 years vs. 39.9 years) [22]. A number of studies in the available literature illustrate the prevalence of CVD in COVID-19 patients, which are described in Table 1. In the studies reported from China, HT was present in 15.0%–34.7%, DM in 7.4%–20.0%, and CVD in 8.7%–15% [3,4,7,8,9,27,42,43]. Most recently, an early report from New York City suggested that the incidence of HT (50.1%) and DM (25.2%) can be higher in U.S. cohorts [34]. A data from 22,512 patients with COVID-19 in the Italian population has demonstrated that 355 patients died in this cohort and 30% of them had ischemic heart disease, 35.5% had DM, 24.5% had atrial fibrillation, and 9.6% had a history of stroke [33]. The high proportion of older patients with COVID-19 in Italy (patients 70 years or older; 37.6% in Italy vs. 11.9% in China) was considered the main determinant of the increased morbidity and mortality. However, the data was derived from the early phase of the pandemic, and trends may change in the future.

Altogether, the increased presence of CVD holds true for COVID-19 patients, most notably among those with severe disease. Currently, the mechanism of these associations remains unclear. Potential explanations include CVD being more prevalent in those with advancing age, a functionally impaired immune system, increased levels of ACE2, or a predisposition to COVID-19 for those with CVD [44].

### 4.2. Outcomes of COVID-19 Patients with Pre-Existing Cardiovascular Disease

A number of studies in the available literature suggest an association between pre-existing CVD and severe COVID-19 (Table 1). In a cohort of 191 hospitalized patients with COVID-19, nonsurvivors were found to have higher incidence of DM (31% vs. 14%, *p* = 0.0051), HT (48% vs. 23%, *p* = 0.0008), and coronary artery disease (CAD) (24% vs. 1%, *p* < 0.0001) than survivors [7]. Analysis of an outpatient and inpatient cohort of 1099 patients with COVID-19 identified that patients who had severe disease were likely to have an increased rate of any coexisting disorders (38.7% vs. 21.0%), DM (16.2% vs. 5.7%), HT (23.7% vs. 13.4%), CAD (5.8% vs. 1.8%), and cerebrovascular disease (2.3% vs. 1.2%) [27]. Increased case fatality rates in the previously referenced analysis of 44,672 confirmed COVID-19 cases from Wuhan, China were noted in patients with CVD (10.5%), DM (7.3%), and HT (6.0%), all remarkably higher than the overall case fatality rate of 2.3% [32].

Cardiovascular risk factors are increasingly recognized to overlap with pathways that regulate immune function. Aging is the strongest risk factor for CVD and its effect on the immune system [45] may be crucial for the severity of COVID-19. Chronic diseases such as HT and DM also correspond with elevated risk of incident CVD and attenuate innate immune response [12]. For instance, metabolic disorders in DM patients may dysregulate immune function by impairing macrophage and lymphocyte function [46] and may confer increased susceptibility to disease complications. In sum, prevalent CVD may be a marker of accelerated immunologic aging/deregulation and relate indirectly to COVID-19. An increased rate of adverse CVD events following COVID-19 infection might also play a role in prognosis, similar to other viral infections, such as influenza [47,48].

## 5. Cardiovascular Complications Following COVID-19

**Figure 1:**
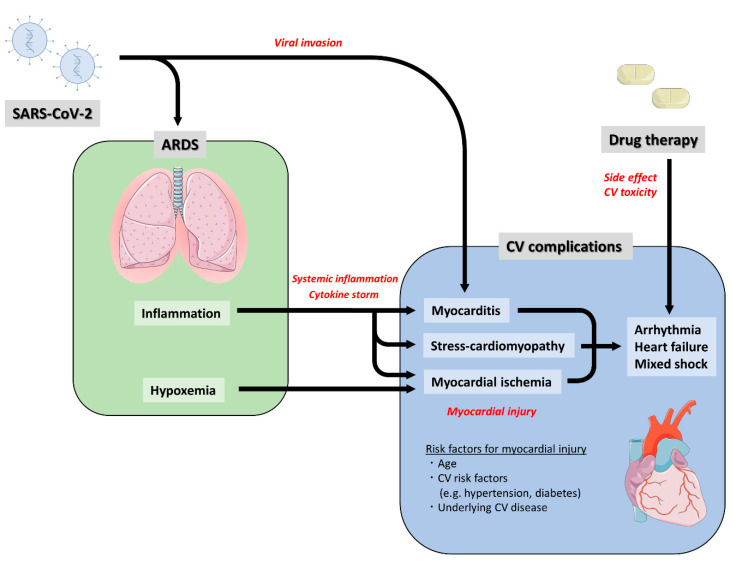
Potential Mechanisms of Cardiovascular Complication Caused by COVID-19. ARDS, acute respiratory distress syndrome; COVID-19, coronavirus disease 2019; CV, cardiovascular. Images were adapted from smart.servier.com by Kensuke Matsushita, 2020, https://creativecommons.org/licenses/by/3.0/ [51].

During prior influenza epidemics, more patients died of CV causes than pneumonia/influenza causes [49]. Given the high inflammatory burden of COVID-19, significant CV complications with COVID-19 infection are expected. Previous reports have suggested that COVID-19 leads to CV complications or deterioration of pre-existing CVD [7,8,20,42,50]. The potential CV complications following COVID-19 are described in Table 2 and Figure 1.

### 5.1. Myocardial Injury

Elevated cardiac biomarkers have been identified in COVID-19 patients, especially in those with severe conditions. Of note, recent case reports highlighted cardiac involvement as a complication associated with COVID-19, even without respiratory symptoms [30,52]. Myocardial injury, defined as elevated cardiac biomarkers, was found in five of the first 41 patients (12%) with COVID-19 in Wuhan, which mainly manifested as an increase in high-sensitivity cardiac troponin I (hs-cTnI) levels (>28 pg/mL) [8]. In this study, the incidence was significantly higher in patients with ICU care than those without (31% vs. 4%, *p* = 0.017).

Consistently, a retrospective, multicenter study including 191 patients with COVID-19 illustrated that elevated hs-cTnI levels were evidenced in 17% of the whole cohort and those myocardial injuries mainly occurred in nonsurvivors (59% vs. 1%, *p* < 0.0001) [7]. A meta-analysis of six studies with 1527 COVID-19 patients found that at least 8% of the patients suffered from acute myocardial injury and the risk of myocardial injury was 13-fold higher in severe patients than nonsevere patients [20].

Myocardial injury can occur due to nonischemic myocardial process or myocardial ischemia. As a nonischemic myocardial factor, myocarditis and stress cardiomyopathy may make a significant contribution in acute myocardial injury of COVID-19 patients. In SARS, Oudit et al. demonstrated that SARS-CoV viral ribonucleic acid (RNA), macrophage infiltration, and myocardial damage were detected simultaneously in 35% of autopsied human heart samples from SARS patients [53]. They confirmed their findings by inducing pulmonary infection with human SARS-CoV in mice, which led to ACE2-dependent myocardial infection in the heart. Likewise, an acute myocarditis in MERS has also been proved by cardiac magnetic resonance image [54]. In line with these findings, a small study including 41 patients with COVID-19 suggested that patients who were clinically severe or critical had an increase of troponin I levels as well as low density of epicardial adipose tissue detected by CT, indicating cardiac inflammation [55]. An early report from China demonstrated that the late increase of hs-cTnI in nonsurvivors (day 16 after disease onset) showed a similar trend to inflammatory markers, including D-dimer, ferritin, IL-6, and lactate dehydrogenase. This kinetics may reflect cytokine storms and systemic inflammatory response syndrome, which provide a possible mechanism for acute myocardial injury due to myocarditis or stress-cardiomyopathy [44,56]. A recent case report by Tavassi et al. suggested that the heart can be directly involved in the viral infection by SARS-CoV-2 [57]. Endocardial biopsy from a COVID-19 patient with acute myocardial injury demonstrated low-grade myocardial inflammation and viral particles in interstitial cytopathic macrophages and their surroundings, but not in cardiomyocytes. In addition, another report by Meyer et al. described a typical case of takotsubo myocardiopathy in COVID-19 patient with acute myocardial injury [58]. Altogether, these findings suggest that an acute myocardial injury in SARS-CoV-2 infection may occur as the disease severity advance by cytokine storms and systemic inflammation or viral infection to the myocardium. Several COVID-19 cases were reported to have cardiac symptoms at admission and diagnosed as fulminant myocarditis [59,60]. However, based on current available, but limited, data, the incidence of isolated fulminant myocarditis appears to be low.

Lessons from the previous influenza epidemics suggest that viral infections can trigger acute coronary syndromes [61]. In a limited study of 75 patients hospitalized with SARS, acute myocardial infarction was the cause of death in two of five fatal cases [26]. Although reports of type 1 myocardial infarction in the setting of COVID-19 are yet to be published, the profound inflammatory response and hemodynamic changes associated with such severe disease may confer an increased risk for atherosclerotic plaque rupture in susceptible patients [7,12]. Moreover, the imbalance of oxygen supply and demand in the heart (type 2 myocardial infarction) may also play a partial role in the myocardial ischemia of COVID-19 patients. Several studies indicated that acute myocardial injury was observed in patients who required oxygen supports, including invasive ventilation and/or ECMO [7,8,9]. Those patients may have hypoxemia or hypotension along with intense systemic inflammation, which may cause an oxygen supply and demand imbalance in the heart [20,42,62], especially when underlying CAD exists. Two recent clinical studies clarified the characteristics of patients having acute myocardial injury. In one cohort of 187 patients from China, 52 patients (28%) experienced troponin T elevation, which was related to the presence of underlying CAD (32.7% vs. 3.0%, *p* < 0.001), cardiomyopathy (15.4% vs. 0%, *p* < 0.001), and CV risk factors. Likewise, another cohort study including 416 Chinese patients suggested that chest pain at admission (13.4% vs. 0.9%, *p* < 0.001), CAD (29.3% vs. 6.0%, *p* < 0.001), and chronic heart failure (14.6% vs. 1.5%, *p* < 0.001) were more common in patients with myocardial injury than those without myocardial injury. Although there are few pieces of evidence to establish a direct association between myocardial injury and CV comorbidities, it is rational to presume that patients with CAD or heart failure are susceptible to cardiac injury, and once such patients are infected with severe pneumonia, myocardial ischemia or cardiac dysfunction are more likely to occur, ultimately leading to a sudden deterioration. Microvascular thrombosis in small coronary vessels due to disseminated intravascular coagulation is another potential but unproven mechanism that may contribute to myocardial injury [63]. Another important mechanism is that troponin levels can also be exacerbated in patients with renal insufficiency due to delayed excretion, which is common in patients with advanced disease.

In sum, myocardial injury in COVID-19 appears common but various mechanisms seem to overlap in individual cases. Given the frequency and nonspecific nature of abnormal troponin results among patients with COVID-19, the American College of Cardiology states that “abnormal troponin should not be considered evidence for an acute myocardial infarction without corroborating evidence” [64]. Comprehensive assessments by electrocardiograms, echocardiograms, magnetic resonance image [52,65], and other cardiac biomarkers, such as brain natriuretic peptide (BNP), may help detect early warnings and improve clinical decision-making. Unfortunately, until now, no specific treatments have been recommended for acute myocardial injury in patients with COVID-19. However, it may be reasonable to triage patients with COVID-19 according to the presence of underlying CVD and the evidence of myocardial injury for prioritized treatment and even more aggressive treatment strategies.

### 5.2. Decompensated Heart Failure and Mixed Shock

The prevalence of new-onset heart failure during COVID-19 associated hospitalization was reported to be 23% and was more common in nonsurvivors compared to survivors (52% vs. 12%, *p* < 0.0001) [7]. Moreover, the first description of critically ill patients from the United States indicated that 33% of the ICU patients developed cardiomyopathy [50]. Most recently, Fried et al. described three cases of COVID-19 infection with acute systolic heart failure [30], suggesting that isolated myocarditis, myocarditis secondary to cytokine storm, stress-cardiomyopathy, and hypoxemia on underlying cardiovascular disease could be considered as potential mechanisms of left ventricular dysfunction. However, given the scarce availability of data regarding this topic, it remains unclear whether the acute heart failure following COVID-19 is mainly due to exacerbation of pre-existing cardiac dysfunction or new-onset cardiomyopathy.

Since imaging features of congestive heart failure in X-ray or CT resemble to those of ARDS (ground-glass opacity and bilateral pulmonary infiltration), echocardiography and serum BNP may help clarify the diagnosis [12]. In particular, COVID-19 infection can cause acute decompensated heart failure when underlying cardiovascular disease exists and may lead to mixed shock [30]. Invasive hemodynamic monitoring using pulmonary artery catheterization can be helpful to manage the cardiac component of shock in such cases.

According to the WHO interim guidelines, ECMO can be considered in COVID-19 patients with refractory hypoxemia despite lung protective ventilation [66]. In patients with MERS-CoV infection, ECMO was associated with reduced mortality in a small cohort study [67]. The role of ECMO in the management of COVID-19 remains unclear at this point. Particularly, it is crucial to determine whether to choose venovenous or venoarterial ECMO cannulation in the context of mixed presentations of ARDS with systolic heart failure. Judgement will be needed to decide when and how ECMO can be worthwhile, by understanding the extent of cardiac function and many other factors. Lastly, providing ECMO during outbreaks of emerging infectious diseases faces unique challenges. Careful planning, judicious resource allocation, and training of personnel to provide complex therapeutic interventions while adhering to strict infection control measures are all crucial components of an ECMO action plan [39].

### 5.3. Cardiac Arrhythmia

High influenza activity has been reported to increase the risk of ventricular arrhythmia [68]. In a study of 121 SARS patients, tachycardia was the most common finding (72%), and other complications were hypotension (50%), bradycardia (15%), and transient paroxysmal atrial fibrillation in only one patient [69]. However, most of these patients were asymptomatic, and these conditions were mostly self-limiting. In hospitalized COVID-19 patients, cardiac arrhythmia was reported in 16.7% of 138 patients and was more common in ICU patients compared to non-ICU patients (44.4% vs. 6.9%) [4]. A recent cohort study reported by Guo et al. described that higher incidence of ventricular tachycardia or ventricular fibrillation was observed in patients with myocardial injury than those without myocardial injury, suggesting a strong relationship between cardiac involvement and arrhythmia in patients with COVID-19 [42]. In addition, high prevalence of arrhythmia in patients with severe disease might be, in part, attributable to metabolic disarray, hypoxemia, or neurohormonal or inflammatory stress in the setting of viral infection [12].

### 5.4. Venous Thromboembolism

Clotting and development of coagulopathy appeared as a noxious complication in severe and critical COVID-19 patients [27,70]. Early reports of critically ill patients with coagulopathy showed poor prognostic features. The first hint suggesting that thrombus formation during severe COVID-19 could increase mortality was reported by Zhou et al. [7]. D-dimer levels over 1 μg/L at admission predicted an 18-fold increase in odds of dying among 191 COVID-19 patients. Following several similar reports, the International Society of Thrombosis and Haemostasis promptly proposed an interim guidance on recognition and management of coagulopathy [71].

In a previously published necropsy series comprising a total of eight SARS patients, pulmonary thromboemboluses were found in four patients and three had deep vein thrombosis (DVT) [72]. However, given the scarce availability of data on this topic, the pathophysiology of venous thromboembolism (VTE) among SARS patients have not been fully characterized. Early case reports [73,74,75] reporting DVT and/or acute pulmonary embolism (APE) in COVID-19 patients paved the way for more comprehensive insights of thrombotic events in larger cohorts. To date, only one retrospective registry cohort of 25 COVID-19 patients in China with CT pulmonary angiography suggested a high rate of thrombotic events, with 10 patients (40%) with APE. With regards to initial sensitivity of RT-PCR at initial presentation that ranges between 60% and 71% [76,77], screening CT for the identification of COVID-19 has been extensively performed. However, CT are currently performed without medium contrast injection, which avoids large scale description of the VTE burden.

COVID-19-infected patients are likely at increased risk of VTE. However, increased coagulopathy abnormalities and thrombotic susceptibility in COVID-19 patients are far beyond the scope of this review. Higher thrombotic burden in the acute phase of COVID-19 relies on proinflammatory cytokine/chemokine release [78,79], increased endothelial dysfunction/damage, and potential sepsis induced coagulopathy development in severe cases, all promoting coagulation activation. Recent insights found that severe lung inflammation and impaired pulmonary gas exchange in COVID-19 has been associated with upregulation of proinflammatory cytokine release and increased endothelial dysfunction [56]. Furthermore, recent evidence support the hypothesis that the endothelium is a key target organ of COVID-19 [80,81]. Endothelial cell activation/damage due to the virus binding to the ACE2 receptor promotes acute inflammation and hypercoagulation, which may be of paramount importance to explain the high thrombotic burden observed [82]. Finally, severe patients with prolonged immobilization are also inherently at high risk for VTE.

APE may present with clinical deterioration, such as unexplained hemodynamic instability and hypoxemia. Elevated cardiac biomarkers and ST-T abnormalities may be detected in some cases [83,84], which can also be observed in other cardiovascular complications. The only widely available treatment for VTE is prophylactic dose low molecular heparin which could be considered in all patients who require hospital admission for COVID-19, in the absence of any contraindications [12,71]. The real incidence of VTE remains unknown in the sparse literature and is probably underestimated because of asymptomatic presentation and/or the lack of systematic imaging. Indeed, routine imaging according to a defined D-dimer threshold has been proposed in some centers such as ours. Special attention needs to be paid to the danger of VTE associated with COVID-19 infection in clinical practice.

### 5.5. COVID-19 and RAAS Inhibitors

ACE2, a surface molecule, is localized in various human organs including oral and nasal mucosa, nasopharynx, lung, and heart [85,86,87]. In renin-angiotensin-aldosterone system (RAAS), ACE2 catalysis the conversion of angiotensin II to angiotensin 1-7, which acts as a vasodilator and exerts protective effects in the CV system [88]. Moreover, ACE2 has been found to be protective from acute lung injury and myocardial injury in animal models [86,89]. As previously shown for SARS-CoV, SARS-CoV-2 similarly utilizes ACE2 for viral cell entry [17] and after the initial engagement of SARS-CoV-2 S protein, there is subsequent downregulation of ACE2 abundance [90]. Dysregulated ACE2 may result in unopposed angiotensin II accumulation and local RAAS activation, which can exacerbate lung and myocardial injuries induced by the viral infection.

There has been a growing concern whether the increased incidence of mortality or myocardial injury in COVID-19 patients with pre-existing CV disorders (HT, DM, and CAD) is due to the use of ACE-inhibitor (ACE-I) and angiotensin-receptor blocker (ARB) [90,91,92]. A question arose whether RAAS inhibition may increase risk of adverse outcomes of COVID-19 through upregulation of ACE2 and increase viral load. In animal models, increased expression and activity of ACE2 in various organs including the heart were found in connection with ACE-I and ARBs administration [93,94]. In contrast to animal experiments, there are few studies in humans regarding the effects of RAAS inhibition on ACE2 expression, showing conflict results [95,96,97]. Moreover, data showing the effects of ACE-I, ARBs, and other RAAS inhibitors on lung-specific expression of ACE2 in experimental animal models and in humans are lacking. Furthermore, there is no evidence providing a causal relationship between ACE2 activity and increased viral load in a critical way. On the contrary, there is abundant evidence of the mortality-lowering effects of RAAS inhibitors in CV disease. Abrupt withdrawal of those drugs in high-risk patients, including those who have heart failure or prior myocardial infarction, may result in clinical instability and adverse outcomes. Consistently, a recent propensity-adjusted retrospective study of 1128 patients with HT and COVID-19 evidenced a reduced mortality rate in patients with ACE-I and ARB therapies [98]. In response, a number of different societies [99,100,101] suggest patients to continue their current ACE-I and ARBs therapies. Two trials of losartan are being conducted among COVID-19 patients who are naïve to RAAS inhibitors and are either hospitalized (NCT04312009) or not hospitalized (NCT04311177).

## 6. Drug Therapy and Cardiovascular Side Effects

Similar to SARS [102] and MERS [22], no specific drug treatment exists for COVID-19 and supportive treatment is the mainstay of management. However, numerous studies are underway to develop vaccines and antivirals to control COVID-19.

The first randomized control trial using the combination protease inhibitor lopinavir/ritonavir, which has been used in HIV, failed to show significant reduction in 28-day mortality or diminish throat viral RNA detectability compared to standard care [103]. Remdesivir is an agent that was originally developed to treat Ebola and has been effective in inhibiting SARS-CoV-2 in vitro by interrupting RNA replication [104]. In a recent single-arm study including 53 hospitalized patients with COVID-19, improvement of oxygen-support status was observed in 68% of patients treated by remdesivir. Several ongoing randomized trials were designed to test this drug on moderate and severe COVID-19 patients (NCT04257656, NCT04252664, NCT04292899, NCT04292730). Chloroquine, which has been used as an antimalarial agent, blocks SARS-CoV-2 cell entry in vitro by increasing the endosomal pH required for virus/cell fusion [104]. A small single-arm study from France has tested the effect of hydroxychloroquine (an analogue of chloroquine) and azithromycin on the respiratory viral loads in patients with COVID-19 [105]. Patients treated with hydroxychloroquine showed a significant reduction in viral carriage by day 6 compared to the control group. Several larger size RCTs are planned to confirm the beneficial effects of this drug (NCT04325893, NCT04318444). Camostat mesylate, an inhibitor of the cellular serine protease TMPRSS2, can block the ACE2-dependent entry of SARS-CoV-2 into host cells [17]. This drug is clinically available in Japan and is a promising agent to be further tested. Antiviral medications used for influenza (oseltamivir, arbidol, and favipiravir) have been applied, without clinical efficacy data available. Lastly, methylprednisolone is another drug under investigation (NCT04323592) that is currently being used to treat severe cases of COVID-19 that are complicated by ARDS [7,8].

It is noteworthy that several therapies have potential interaction with CV medications and CV toxicities. Lopinavir/ritonavir may prolong PR and QT interval, and caution should be taken in patients with underlying CVD including pre-existing conduction abnormalities or those taking other QT prolonging drugs [106]. Lopinavir/ritonavir can also influence the activity of P2Y_12_ inhibitors through CYP3A4 inhibition, which results in decreased serum concentrations of active metabolites of clopidogrel and prasugrel and increased serum concentration of ticagrelor. Cardiac side effects of chloroquine/hydroxychloroquine are rarely reported, but in some cases can be severe and irreversible [107,108]. It is thought to be due to the inhibition of lysosomal enzyme in the myocyte which leads to conduction abnormalities and cardiomyopathy [108]. Careful QT monitoring is recommended especially when a combination of hydroxychloroquine and azithromycin is used [109]. Lastly, the use of corticosteroid is known to cause fluid retention, electrolyte derangement, and hypertension as direct CV effects.

With the current COVID-19 pandemic, numerous RCTs have been launched around the world. By participating in an RCT, patients may benefit from a unique opportunity to directly contribute to the discovery of a new therapy. However, careful monitoring of CV complications is often required to prevent patient global deterioration.

## 7. Prognosis of Survivors Undergoing Respiratory Virus Infection

Residual systemic inflammatory and procoagulant activity can be observed in survivors of hospitalization for pneumonia long after resolution of the index infection [56]. Nevertheless, long-term follow-up data concerning the survivors of respiratory virus epidemics are scarce. An observational study previously demonstrated that hospitalization for pneumonia was associated with increased short-term and long-term risk of CVD, suggesting that pneumonia may be an important risk factor for CVD [110]. In order to increase our understanding, serial follow-up studies amongst the survivors of COVID-19 are sorely needed.

## 8. Conclusions

COVID-19, caused by SARS-CoV2, has resulted in considerable morbidity and mortality worldwide and has become an emerging health threat. Underlying CVD is a common condition among patients hospitalized with COVID-19 and is associated with a higher risk of severe disease and morality. Myocardial injury and myocardiopathy are present in a considerable number of critical cases and patients with pre-existing CAD or underlying heart failure seem to be susceptible to myocardial injury. Despite the theoretical concerns and uncertainty regarding the effect of RAAS inhibitors on ACE2 and the way in which these drugs affect the severity of COVID-19, RAAS inhibitors are recommended to be continued based on the available evidence at this time. A large number of prospective RCTs and cohort studies are ongoing, but none have provided proven clinical efficacy to date.

## Figures and Tables

**Table 1:** Prevalence of Cardiovascular Risk Factors and Underlying Cardiovascular Disease in COVID-19 Patients.

	Age	Cardiovascular Disease	Coronary Artery Disease	Hypertension	Diabetes	Chronic Kidney Disease	Current Smoker
Chen et al. 2020 (*n =* 99) [3]	55 ± 13	40 (40%) *	-	-	-	-	-
Wang et al. 2020 (*n =* 138) [4] ICU vs. Non-ICU	56 (42–68)	20 (14.5%) 25.0% vs. 10.8%	-	43 (31.2%) 58.3% vs. 21.6%	14 (10.1%) 22.2% vs. 5.9%	4 (2.9%) 5.6% vs. 2.0%	-
Huang et al. 2020 (*n =* 41) [8] ICU vs. Non-ICU	49 (41–58)	6 (15%) 23% vs. 11%	-	6 (15%) 15% vs. 14%	8 (20%) 8% vs. 25%	-	3 (7%) 0% vs. 11%
Zhou et al. 2020 (*n =* 191) [7] Non-Survivor vs. Survivor	56 (46–67)	-	15 (8%) 24% vs. 1%	58 (30%) 48% vs. 23%	36 (19%) 31% vs. 14%	2 (1%) 4% vs. 0%	11 (6%) 9% vs. 4%
Guan et al. 2020 (*n =* 1099) [27] Severe vs. Non-Severe	47 (35–58)	-	27 (2.5%) 5.8% vs. 1.8%	165 (15.0%) 23.7% vs. 13.4%	81 (7.4%) 16.2% vs. 5.7%	8 (0.7%) 1.7% vs. 0.5%	137 (12.6%) 16.9% vs. 11.8%
Ruan et al. 2020 (*n =* 150) [39] Died vs. Discharged	-	13 (8.7%) 19% vs. 0%	-	52 (34.7%) 43% vs. 28%	25 (16.7%) 18% vs. 16%	2 (1.3%) 3% vs. 0%	-
Guo et al. 2020 (*n =* 187) [38]	59 ± 15	-	21 (11.2%)	61 (32.6%)	28 (15.0%)	6 (3.2%)	-
Shi et al. 2020 (*n =* 416) [9]	64 (21–95)	-	44 (10.6%)	127 (30.5%)	60 (14.4%)	14 (3.4%)	-
Goyal et al. 2020 (*n =* 393) [34]	62 (49–74)	-	54 (13.7%)	197 (50.1%)	99 (25.2%)	-	20 (5.1%)

Values are *n* (%), mean ± SD, or median (interquartile range); COVID-19, coronavirus disease 2019; ICU: intensive care unit; SD, standard deviation. * Composite of cardiovascular and cerebrovascular diseases.

**Table 2:** Prevalence of Cardiovascular Complications, Acute Respiratory Distress Syndrome (ARDS), and Extracorporeal Membrane Oxygenation (ECMO) in COVID-19 Patients.

	Age	Myocardial Injury	Arrhythmia	Heart Failure	Shock	ARDS	ECMO
Chen et al. 2020 (*n =* 99) [3]	55 ± 13	-	-	-	4 (4%) *	17 (17%)	3 (3%)
Wang et al. 2020 (*n =* 138) [4] ICU vs. Non-ICU	56 (42*–*68)	10 (7.2%) 22.2% vs. 2.0%	23 (16.7%) 44.4% vs. 6.9%	-	12 (8.7%) 30.6% vs. 1.0%	27 (19.6%) 61.1% vs. 4.9%	4 (2.9%) 11.1% vs. 0%
Huang et al. 2020 (*n =* 41) [8] ICU vs. Non-ICU	49 (41*–*58)	5 (12%) 31% vs. 4%	-	-	3 (7%) 23% vs. 0%	12 (29%) 85% vs. 4%	2 (5%) 15% vs. 0%
Zhou et al. 2020 (*n =* 191) [7] Non-Survivor vs. Survivor	56 (46*–*67)	33 (17%) 59% vs. 1%	-	44 (23%) 52% vs. 12%	38 (20%) * 70% vs. 0%	59 (31%) 93% vs. 7%	3 (2%) 6% vs. 0%
Guan et al. 2020 (*n =* 1099) [27] Severe vs. Non-Severe	47 (35*–*58)	-	-	-	12 (1.1%) * 6.4% vs. 0.1%	37 (3.4%) 15.6% vs. 1.1%	5 (0.5%) 2.9% vs. 0%
Guo et al. 2020 (*n =* 187) [38] Elevated vs. Normal TnT	59 ± 15	52 (27.8%) NA	11 (5.9%) ^†^ 17.3% vs. 1.5%	-	-	46 (24.6%) 57.7% vs. 11.9%	-
Shi et al. 2020 (*n =* 416) [9] With vs. Without myocardial injury	64 (21*–*95)	82 (19.7%) NA	-	-	-	97 (23.3%) 58.5% vs. 14.7%	-

Values are *n* (%), mean ± SD, or median (interquartile range); ARDS: acute respiratory distress syndrome; ECMO: extracorporeal membrane oxygenation; ICU: intensive care unit; NA: not applicable; TnT: Troponin T. * septic shock. ^†^ ventricular tachycardia/ventricular fibrillation.
